# Dental care for persons with disabilities: discretion on the frontline

**DOI:** 10.11606/s1518-8787.2023057005318

**Published:** 2023-09-29

**Authors:** Joana Danielle Brandão Carneiro, Ana Paula Chancharulo de Moraes Pereira, Aylene Bousquat, Paulo Frazão

**Affiliations:** I Universidade de São Paulo Faculdade de Saúde Pública Departamento de Política, Gestão e Saúde São Paulo SP Brasil Universidade de São Paulo. Faculdade de Saúde Pública. Departamento de Política, Gestão e Saúde. São Paulo, SP, Brasil; II Universidade Estadual da Bahia Departamento de Ciências da Vida Bahia BA Brasil Universidade Estadual da Bahia. Departamento de Ciências da Vida. Bahia, BA, Brasil

**Keywords:** Public Policy, Dental Care, Disabled Persons

## Abstract

**OBJECTIVE:**

To depict the influence of discretionary actions exercised by frontline professionals and organizations on the implementation of diverse modalities of access to specialized dental care within the Care Network for Persons with Disabilities.

**METHODS:**

A case study conducted in two Brazilian health regions characterized by distinct means of access to specialized dental care employing documentary analysis and interviews with key stakeholders across the period spanning from July to December 2019.

**RESULTS:**

In the referenced access region, there was a notable centrality of Primary Health Care (PHC) in caregiving, wherein planning and assessment were integral components of institutional routines. Where spontaneous demand scheduling was accepted, sporadic exchanges of information were evident between PHC units and specialized facilities. The coordination role in caregiving was not vested in PHC teams, and activities such as planning and assessment were not assimilated into organizational routines.

**CONCLUSIONS:**

The implementation of policies for specialized dental care for persons with disabilities relied on the coordination furnished by PHC and the orchestration of planning and assessment endeavors aimed at establishing an integrated care network. This implementation proved subject to the discretionary authority of frontline professionals and organizations, highlighting the significant role of relational and institutional environments in the context of public policy implementation within a decentralized and regionalized healthcare system.

## INTRODUCTION

Over the past three decades, several strategies of decentralization and regionalization of healthcare services have gained traction^1^. The implementation of health policies has become contingent upon diverse organizations and a multitude of public-private agreements at the local level. Within this context, discretion—a pivotal concept in the theory of street-level bureaucracy^2^—, has been scrutinized across various governing dimensions of decision-making, encompassing ethical values, rules, normative provisions, as well as professional and organizational aspects that translate into the expected roles of frontline professionals and organizations^3^.

The ways in which frontline organizations/professionals provide benefits and sanctions contribute to shaping and circumscribing individuals’ lives, either by broadening or restricting opportunities. Enlisted as public utility services providers and public policy implementers, frontline professionals, along with the organizations dependent upon their operational costs, often find themselves at the strained core between the demands of service recipients seeking greater effectiveness and responsiveness, and the expectations of citizens, urging for heightened efficiency and efficacy from the organizations tasked with provisioning public services^2^.

Agents’ discretion in the process of implementing a given public policy may vary in scope, contingent upon the degree of policy structure/detail and the comprehensiveness and ambiguity of rules upheld within the organizations where they belong^4^. While the exercise of political discretion may weaken or bolster the general interest and legitimacy of a program, administrative discretion is perceived as a spectrum of choices existing within a set of parameters delineated through organizational rules. Frontline organizations/professionals utilize their discretion to form arrangements blending different dimensions aimed at attaining politically and socially desired outcomes within a legitimately defined direction. These arrangements could elucidate disparities in the frontline implementation of public policy^3^. Such arrangements are not solely born from interpersonal interactions but can result from the interplay of a network comprising diverse professionals and frontline organizations operating either in isolation or collaboratively^5^.

The Brazilian literature lacks works that incorporate analytical models of health policy implementation and street-level bureaucracy. Some studies delve into the discretionary power of community health agents in implementing primary care policy actions^6^, as well as the role of street-level bureaucrats in executing the policy of water fluoridation at public supply plants in small Brazilian municipalities^7^. Studies investigating the discretion of street-level bureaucracy in the implementation of *redes de atenção à saúde* (RAS – health network policies) remain largely unexplored, making it opportune to investigate specialized dental care access within the framework of the Care Network for Persons with Disabilities (*Rede de Cuidados à Pessoa com Deficiência* – RCPD). Access can be defined by the various strategies adopted by organizations to facilitate users’ utilization of needed services^8^.

In the first decade of the 21st century, the enactment of the National Policy for Persons with Disabilities (*Política Nacional da Pessoa com Deficiência* – PNPD) and the National Oral Health Policy (*Política Nacional de Saúde Bucal* – PNSB) in Brazil propelled the integration of basic dental care structures, with oral health teams working in the primary health care (PHC) network, as well as specialized care, via dental specialty centers (DSC), to provide more complex ambulatory treatments for all in need, including persons with disabilities (PwD)^9^.

The RCPD, established within the Brazilian Unified Health System (SUS) in 2012, stimulates intergovernmental coordination to ensure the access of PwD to various specialized treatments, via regional agreements and governance involving state/municipal authorities of a specific health region. Safeguarding the right to dental treatment and comprehensive care is no simple task and calls for harmonious coordination among different points of care within the RCPD^9^, wherein the PHC should stand out as the healthcare coordinator^8^.

A study encompassing 930 DSCs revealed that 85% of the units were municipally managed and 10% of them did not provide care for PwD, despite it being mandatory. User access was referred by the PHC in just over half of the specialized units. A significant share (42.7%) allowed for the scheduling through spontaneous demand^10^, but no study has explored this contrasting situation. It is conceivable that the implementation of this public policy hinges on the actions of frontline professionals and organizations. Producing scientific information on the interplay between PHC and specialized services aimed at PwD could enhance the understanding of progress and challenges in the development of integrated healthcare networks.

This study aims to depict the role of frontline professionals and organizations concerning the PHC and planning/assessment activities to grasp the exercise of discretion in implementing different means of specialized dental care access in the RCPD across two Brazilian health regions.

## METHODS

A case study^11^ was conducted in two regions with disparate access to specialized dental care. They were selected based on documentary data and interviews performed in a broader study^a^ approved by the Research Ethics Committee Faculdade de Saúde Pública of Universidade de Sá0 Paulo, nº. 3,441,243.

Access to specialized care was referenced in the healthcare region of São José do Rio Preto (Region A), while access was mixed in Salvador (Region B). In Region B, a significant share of users accessed the specialized unit freely without the need for formal referral, subject to its own rules.

The two regions were intentionally chosen due to their similar socioeconomic characteristics and services provided. In a typology conducted by Viana et al.^12^, they were classified as regions with high socioeconomic status, as well as a high supply and complexity of health services. Moreover, both regions had specialized rehabilitation centers (SRC), dental specialty centers, municipal and/or philanthropic specialty services with rehabilitation actions for users (such as the *Associação de Pais e Amigos dos Excepcionais*, the *Associação de Assistência à Criança Deficiente*, both NGOs), and educational institutions (universities, colleges, and medical and/or multi-professional residency programs).

Data collection took place between July and December 2019 and was conducted by trained professionals. Five specialized dental care units for PwD and four primary care units under municipal management in the two regions agreed to participate in the research. Of the five specialized units, four were municipally managed (one in Region A and three in Region B), and one was state-managed (Region B). Nine frontline professionals were interviewed, represented in this study by their managers, and considered key actors within the organizations. When a unit did not have a manager, the key informant was the dentist responsible for the care of PwD.

Structured (questionnaires) and semi-structured (scripts) instruments were used for data collection. In this study, respondents’ answers regarding the frequency of PwD access to specialized units through the PHC, the prioritization criteria adopted, challenges for the PHC to act as the main gateway to services, PHC initiatives to coordinate care, and the use of monitoring/assessment instruments, their frequency, and participating actors were analyzed.

Technical reports and minutes of collegial meetings regarding content related to the implementation of the Care Network for Persons with Disabilities at the local-regional level, spanning events from 1988 to 2019, were examined to identify normative provisions and aspects related to PHC organization and planning/assessment activities. To achieve this, decrees, laws, and regulations related to the topic were consulted on the institutional websites of the regions (Municipal and State Secretariats, State Planning and Management Secretariat), technical reports produced within the health regions, and minutes of meetings of the *Comissão Intergestores Bipartite* (CIB – Bipartite Intermanagement Committee) and *Comissão Intergestores Regional* (CIR – Regional Intermanagement Committee), made publicly available or provided by service technicians. The selection of document excerpts was based on keywords identifying nuclei of meaning^13^ related to the following categories: type of access; attribute of PHC related to care coordination; planning, evaluation, and monitoring.

Access can be defined by the various strategies adopted by organizations to facilitate users’ utilization of needed services. Care coordination refers to the clinical management of cases through the integration of actions and services provided by different units in the care network to meet users’ health needs and aim to reorient the care model. Planning, evaluation, and monitoring concern specific guiding instruments and indicators for the frontline professionals’ work process, as well as the quality of the provided actions^8^.

## RESULTS

The results are presented according to theme categories and regions. Charts 1, 2, and 3 present excerpts from the interviews, grouped into the categories “Access to Specialized Dental Care for PwD,” “The PHC as a Care Coordinator,” and “Planning, Evaluation, and Monitoring,” respectively. Chart 4 provides a synthesis of the results by theme category in Regions A and B. Interviewees were identified as E_R1_, E_R2_, and E_R3_ for Region A, and from E_R4_ to E_R9_ for Region B.

**Chart 1:** Interviewees’ reports grouped under the category “Access to Specialized Dental Care for PwD” for Regions A (referred access) and B (mixed access).

Region A	Region B
“*The Basic Health Unit is the preferred point of entry in the region”* (E_R1_).	“*The point of entry is through referral from the UBS. They seek the UBS, Emergency Care , Emergency Care Unit, or Family Health Strategy (when applicable), and, if the dentist does not provide treatment, they create a document referring the patient to be treated at the DSC”* (E_R4_).
“*I think that, if our DSC is functional today, it’s since basic care started working well and is effective.* “*Otherwise, the DSC can’t be organized if basic care doesn’t work properly, according to the protocol*” (E_R2_)	“*So, the patients are aware that, once they arrive here with their institute card showing their affiliation, along with their medical report stating that they have special needs, they will receive care here without the need of a referral*” (E_R5_).
“*We must work as a network—the DSC, basic, and specialized care. Only then things will work as they should*” (E_R3_).	“*For a patient to enter the unit, they must go through the orientation group* [OG], *which determines whether they fit the profile or not. Once accepted, they are assisted by a group of professionals who assess the users and determine their actual needs and may list them as a priority, or not*” (E_R6_).

PwD: persons with disabilities; UBS: Basic Health Unit; DSC: dental specialty centers.

**Chart 2:** Interviewees’ reports grouped under the category “The PHC as a Care Coordinator” for Regions A (referred access) and B (mixed access).

Region A	Region B
“*We provide matrix support to basic care, conducting training four times a year. And this is agreed upon with basic care*.” (E_R2_).	“*Basic care is quite fragmented. The increasing number of diabetics each day is a clear sign of the failure of basic care*” (E_R7_).
	“[PHC] *coverage is lacking throughout nearly 30% of Salvador*[...] *We really wished primary care coverage would reach it there; I believe many of our problems would be solved if we could achieve greater coverage*” (E_R8_).
	“*Not the special cases* [PwD]. *I handle everything here. I don’t refer them, except for patients who are not special cases* [PwD]*, who are now returning to the UBS*” (E_R8_).

**Chart 3:** Interviewees’ reports grouped under the category “Planning, Evaluation, and Monitoring” for Regions A (referred access) and B (mixed access).

Region A	Region B
“*If you don’t know the regulation, you won’t know how to make the DSC work. And there’s a regulation for everything. The production demanded by the Ministry is ludicrous, some municipalities can’t handle it. So, it’s a matter of knowing the regulation and saying, ’Now you have to make it happen!’*” (E_R2_).	“*There’s no action planning*” (E_R9_).
“*I read the regulation, we read it together, discussed it in team meetings, I brought the regulations and think that’s important. I conveyed the regulations so they would fully understand them*” (E_R2_).	“[They hold team meetings] *at least every 2 months, because of the professionals’ workload (the whole team)*” (E_R8_).
	“*We treat patients, but there are no goals. Usually, the Type III DSC performs 190 basic procedures for PwD, of which at least 50% must be restorative*” (E_R8_).

**Chart 4:** Summary of Results According to the Theme Categories: RCPD in Oral Health (RCPD-SB), in two Brazilian healthcare regions, 2019.

Organization of the RCPD-SB		
Theme Categories	Region A	Region B
Access	Only through PHC referral.	Referenced by the PHC or another specialized service, or through open demand.
The PHC in Care Coordination	Yes. Conducted matrix support on the subject (RCPD), established care pathways, rehabilitation actions, and scheduling of timely appointments available for referred users.	Did not coordinate, particularly due to low population coverage. No counter-reference took place from the DSC to the PHC.
Planning, Monitoring, and Evaluation	Monthly evaluation and monitoring in team meetings, using indicators and a specific instrument created by the DSC management. The steering group worked as a device for sharing information and decisions.	Excluded from the routine of RCPD-SB services. Each service would define its work process and did not always follow the standards established in regulations and ministerial policies. Lacked room for sharing information.

The discussions between different levels of government within the CIB on the management and organization of RCPD started at the end of 2011 in Region A, while in Region B, they were only added to the agenda in 2013. In Region A, where access to specialized dental care only occurred through formal requests issued by a service unit in the network, it was observed that this reference happened when the PHC dentist lacked the necessary conditions to provide adequate treatment. This was also the case in Region B, where access was both through open demand and referral. In this mixed type of access, users could enter freely, without the need for a formal request, and according to the specialized unit’s own rules.

The UBS was the preferred access point in Region A, to access other specialized rehabilitation services included in the RCPD (such as SRC and the *Ambulatório Médico de Especialidade* – AME [Specialty Outpatient Clinics]), being responsible for referring users to specialized services. Access to specialized units located in the main municipality was through the PHC, obligatorily. Cases that did not follow this criterion were admitted for guidance and redirected to the PHC.

Excerpts from documents pointed out that ensuring RCPD accessibility was a recurring theme in meetings of the *Comissão Intersetorial de Atenção à Pessoa com Deficiência* (Intersectoral Committee for the Care of Persons with Disabilities) in Region A. This effort involved the expansion of the availability of sanitary transport and assistance offering for the use of regular buses.

In Region B, there were specialized dental care units managed either by the state or municipalities. It was observed that professionals from the state-affiliated unit did not communicate or interact with dentists working in PHC and specialized units belonging to the municipal network. In practice, that service had an entry point following pre-established rules. On the other hand, the relevance of networking in Region A was recognized to enhance care for PwD and, consequently, RCPD (Chart 1).

The cost of transportation means in Region B and how hearing-impaired PwD were admitted to specialized units in Region A were identified as potential access barriers to services. The main municipality in Region A had a professional interpreter of Libras (Brazilian Sign Language) in place, who acted as a translator during specialized dental consultations through prior scheduling.

It was noticeable that UBSs were not the preferred access point for health services for users in Region B. Their role as care coordinators was weakened by the low potential for population coverage and the fragmentation of care, pointing to the significant challenge of integrating the PHC with other specialized services. During CIB meetings, strategies were proposed for the development of the State Plan, such as establishing care pathways, organizing the flow of services, enrolling family health teams with oral health expertise, and expanding the PHC. However, no changes were observed at the frontline. The counter-referral of users back to the PHC by specialized unit professionals in Region B also did not take place (Chart 2).

The PHC played a central role in health care across Region A, being primarily responsible for the care for PwD. Professionals participated in matrix support on the subject, providing rehabilitation actions and managing to schedule timely rehabilitation appointments for referred users. The DSC’s matrix support agenda with the PHC was planned annually through team meetings. The UBSs which carried out oral health care support in both regions also provided care for PwD. The more complex cases, such as non-collaborative users needing restraint or more individualized intervention, were referred to DSCs.

Planning, evaluation, and monitoring of actions were not a part of the institutional service routine in Region B. As a consequence, the work process was neither monitored nor guided by any pre-established criteria. Only a few units held regular team meetings and monitored indicators. It should be noted that the State Plan of the RCPD envisaged the production, follow-up, and monitoring of information, as well as professional qualification, as a management qualification guideline (Chart 3).

Planning, evaluation, and monitoring were part of the institutional routine of services in Region A, and some managers (such as the DSC’s) developed their own instrument for evaluating and monitoring specific indicators regarding user satisfaction, number of restorative dental procedures, absenteeism proportion, percentage of completed treatments, number of extractions, and amount of PwD served in all specialties, among others. The meetings of the RCPD Steering Group also recognized the importance of both action evaluation and monitoring for the consolidation of the RCPD through the situational diagnosis (number of PwD, main health demands, regional plan development), and the role of management in using action monitoring tools and sharing information in meetings with the entire team.

## DISCUSSION

This case study described the role of professionals and frontline organizations in understanding the exercise of discretion in implementing different forms of access to specialized dental care in the RCPD across two Brazilian health regions.

The findings highlighted distinct characteristics regarding the PHC, planning, and evaluation activities, as well as the access strategy in place. The PHC played a care coordination role in Region A, where access took place through referral, and planning/evaluation were part of the institutional routine of services. In Region B, which worked with mixed access, there was the occasional information exchange between PHCs and specialized units, and the care coordination role was not attributed to the PHC teams. There, planning/evaluation activities were not incorporated into the organizational routine, as guided by PNSB, as each service decided, through its implementers, whether to conduct them or not.

As a result, the exercise of discretion in the way user access was regulated in each region was closely related to the PHC’s role within the care network and the characteristics of specialized units concerning planning/evaluation activities. The distinct outcomes of implementation found in this case study confirmed the idea that effective implementation is usually based on the references implementers embrace to perform their functions and corroborate the notion that the exercise of discretion encompasses different dimensions aimed at achieving politically and socially desired outcomes in a direction legitimately defined by the relational and institutional environment present in the local-regional context, emphasizing both the administrative and political dimensions^3^.

Administrative discretion is understood as the use of strategies to introduce procedural changes^3^. This case study refers to the use of communication channels and shared flows with the help of protocols and other common instruments to promote coordination between PHC units and specialized dental services aspiring to achieve increasing levels of care integration. It also includes the decision to produce both shared planning and evaluation spaces among managers of different programs and services within the same organization, or involving different units of equal or distinct technological density, as the use of discretionary action by public actors calls for governance skills developments^3^. The implementation of protocols and common flows throughout Region A’s network was a characteristic that assisted in the coordination of primary and specialized level organizations, allowing for the adoption of referred access by specialized dental services.

The political dimension concerns the values and references at play in the interaction of actors and the competence to combine them during the exercise of power to make them effective in the achievement of the desired ends^3^. Although we can identify a component related to the individual trajectory of actors, those references are not only produced by individual choices but also engendered by influences derived from relationships established during implementation in specific institutional and relational contexts^1^, in which collaboration among stakeholders can help shorten the path to achieving the intended outcomes^14^.

A policy of general interest related to the construction of an integrated healthcare network was underway in Region A and was mentioned by interviewees from both PHCs and specialized units. On the other hand, in Region B, this construction was not from the actors’ perspective. The material obtained evidenced that communication and interaction between professionals from the units were not structuring resources for care actions. In this relational environment, units operated in an isolated and fragmented logic of providing basic and specialized services.

After the approval of the PNSB guidelines in 2004, over a thousand DSCs were created in Brazil to enable referred access for PwD and the general population to complex care by adhering to the principle of care comprehensiveness^15^. Although the strategy of open demand is generally regarded as negative as a means of accessing specialized services within a care network focused on integrated care delivery, it can be interpreted positively by users who do not resort to the PHC for subsequent consultations. In a relational and institutional context where units do not operate collaboratively or establish reciprocal commitments, and fragmented practices are the rule, resorting to another unit may lead to additional costs, uncertainty, and stress for the user. This gives way to distortions in the implementation of DSCs from the perspective of the public policy analyzed, allowing for their operation to respond to “counter” logic focused on achieving politically and socially desired results, related to specific short-term interests that may lead to a client-focused perspective^16^.

It is worth noting that, since the late 1990s in Brazil, the local level has been responsible for providing primary health care services, while the provision of specialized services depends on regional arrangements between the municipal and state levels. Decentralized systems in federal republics, similar to countries such as Canada and Australia, can represent a significant advantage by allowing cost containment at the central level and granting greater autonomy and responsibility to local governments in addressing the health needs of the population. However, it is also a challenge because municipalities and regions are subject to jurisdictional variations, and fragmentation in coordination, cooperation, and information sharing.^17^.

Examining the performance of community health agents^6^, it was found that activities varied considerably, although they performed the same function and were governed by the same policy. In addition to individual factors, organizational and contextual aspects would also influence the type of activity carried out by the agents in their routine. In this study, despite the services being provided by frontline professionals with backgrounds in the health field and governed by general interests defined by the guidelines of a public policy focused on RCPD implementation, the strategies for accessing specialized dental care were different. Despite similarities in socioeconomic conditions and service availability, the implementation outcomes differed, indicating the significant influence of the relational and institutional environments.

In terms of the limitations of this study, it’s important to highlight that implementation at the frontline is influenced by multiple forces, often competing in the implementation system^14^. The analysis of this study did not encompass the viewpoint of service users. Further research that integrates the perspectives of both professionals and users could enhance and delve deeper into the insights presented here, aiming to investigate the consequences of discretion that may result in inclusion, equity, exclusion, and inequality. Sociocultural differences, the prevalence of PwD, and the number of users in each region were also not considered. Despite these points and the results being derived from a case study, the investigation is innovative in its use of the theory of public policy implementation from a *bottom-up* perspective. It explores analytical domains that are less investigated concerning the relational and institutional aspects that influence the frontline of the investigated public policy.

The implementation of a specialized dental care policy for persons with disabilities is subject to the discretionary power of frontline professionals and organizations. This implies that the relational and institutional environment plays a significant role in the process of implementing public policies within a decentralized and regionalized healthcare system. In such a system, diverse strategies for accessing specialized services are linked to the coordinating role of PHC and the execution of planning and evaluation activities aimed at constructing an integrated healthcare network.

## Footnotes

a “Desafios da implementação da Rede de Cuidado à Pessoa com Deficiência em diferentes contextos regionais: abordagem multidimensional e multiescalar” (MS-SCTIE-DECIT/CNPQ nº 442801/2018-1).
