# Supplementary material for: Dental care for persons with disabilities: discretion on the frontline
Source: Rev Saude Publica. 2023 Sep 29;57:74. doi: 10.11606/s1518-8787.2023057005318 (PMC10547384; doi:10.11606/s1518-8787.2023057005318)
Supplement: Translation

# Translation: Assistência odontológica à pessoa com deficiência: discricionariedade na linha de frente

**Authors:** Joana Danielle Brandão Carneiro, Ana Paula Chancharulo de Moraes Pereira, Aylene Bousquat, Paulo Frazão

**Affiliations:** I Brasil Universidade de São Paulo. Faculdade de Saúde Pública. Departamento de Política, Gestão e Saúde. São Paulo, SP, Brasil; II Brasil Universidade Estadual da Bahia. Departamento de Ciências da Vida. Bahia, BA, Brasil

**Keywords:** Política Pública, Assistência Odontológica, Pessoas com Deficiência

## Abstract

**OBJETIVO:**

Descrever a influência da discricionariedade dos profissionais e organizações da linha de frente na implementação de diferentes formas de acesso à assistência odontológica especializada na Rede de Cuidados à Pessoa com Deficiência.

**MÉTODOS:**

Estudo de caso em duas regiões de saúde brasileiras cujo acesso à assistência odontológica especializada era distinto, com análise documental e entrevista com atores-chave, entre julho e dezembro de 2019.

**RESULTADOS:**

Na região com acesso referenciado, observou-se que a atenção primária à saúde (APS) tinha centralidade no cuidado e o planejamento/avaliação faziam parte da rotina institucional dos serviços. Na região onde o agendamento era possível por demanda espontânea, notou-se trocas episódicas de informação entre as unidades de APS e as especializadas; o papel de coordenação do cuidado não era um atributo das equipes de APS e as atividades de planejamento/avaliação não estavam incorporadas à rotina das organizações.

**CONCLUSÕES:**

A implementação da política de assistência odontológica especializada à pessoa com deficiência se mostrou dependente da coordenação da APS e da condução de atividades de planejamento/avaliação voltadas à construção de uma rede de cuidados integrada e sujeita ao poder discricionário dos profissionais e das organizações da linha de frente, sugerindo que o ambiente relacional e institucional possui um papel importante no processo de implementação de políticas públicas em um sistema descentralizado e regionalizado de saúde.

## INTRODUÇÃO

Nos últimos 30 anos, ganharam impulso diferentes estratégias de descentralização e regionalização de serviços de saúde^1^. A implementação de políticas de saúde passou a depender de diferentes organizações e uma infinidade de acordos público-privados em nível local. Neste contexto, a discricionariedade, enquanto conceito-chave da teoria da burocracia de nível de rua^2^, passou a ser investigada a partir de diferentes dimensões que orientam a tomada de decisão, desde valores éticos, regras e dispositivos normativos, até aspectos profissionais e organizacionais que se manifestarão nos papéis esperados das organizações e profissionais da linha de frente^3^.

As formas pelas quais organizações/profissionais da linha de frente proporcionam benefícios e sanções contribuem para estruturar e delimitar a vida das pessoas, ampliando ou restringindo oportunidades. Como prestadores de serviços de utilidade pública e operadores da política pública, os profissionais da linha de frente, e as organizações que têm seus custos operacionais deles dependentes, podem ser foco de tensões entre as demandas dos destinatários dos serviços, que querem maior efetividade e responsividade, e as demandas de cidadãos, cobrando mais eficácia e eficiência das organizações responsáveis pela oferta dos serviços públicos^2^.

A discricionariedade dos agentes no processo de implementação de uma determinada política pública pode ser mais ampla ou restrita, conforme o grau de estruturação/detalhamento da política e a abrangência/ambiguidade das regras mantidas pelas organizações onde eles operam^4^. Enquanto o exercício da discricionariedade em sua dimensão política pode enfraquecer/fortalecer o interesse geral e a legitimidade do programa, a discricionariedade administrativa é vista como uma gama de escolhas dentro de um conjunto de parâmetros que se manifestam na forma de regras organizacionais. Organizações/profissionais usam sua discricionariedade para fazer arranjos, que mesclam diferentes dimensões voltadas à obtenção de resultados política e socialmente desejados em uma direção legitimamente definida, que podem explicar diferenças na linha de frente da implementação da política pública^3^. Estes arranjos não decorrem só da interação entre pessoas, mas podem ser resultado da interação de uma rede de diferentes profissionais e organizações da linha de frente que atuam isolada ou colaborativamente^5^.

Na literatura brasileira, trabalhos que incorporam modelos analíticos de implementação de políticas de saúde e burocracia de nível de rua são escassos. Há estudos que analisam o poder discricionário de agentes comunitários de saúde na implementação de ações da política de atenção básica^6^, e a atuação de burocratas de nível de rua na implementação da política de fluoretação das águas de abastecimento público em pequenos municípios brasileiros^7^. Estudos sobre a discricionariedade da burocracia do nível de rua na implementação de políticas de redes de atenção à saúde (RAS) são pouco explorados, sendo oportuna a investigação do acesso à assistência odontológica especializada no âmbito da Rede de Cuidados à Pessoa com Deficiência (RCPD). O acesso pode ser definido pelas diferentes estratégias adotadas por organizações para que os usuários consigam utilizar os serviços de que necessitam^8^.

Na primeira década do século XXI, a aprovação da Política Nacional da Pessoa com Deficiência (PNPD) e da Política Nacional de Saúde Bucal (PNSB) no Brasil impulsionaram a articulação da estrutura de cuidados odontológicos básicos, por meio de equipes de saúde bucal na rede de atenção primária à saúde (APS), e especializados, por meio de centros de especialidades odontológicas (CEO), a fim de oferecer tratamentos ambulatoriais mais complexos a todos que deles precisarem, incluindo as pessoas com deficiência (PcD)^9^.

A RCPD, criada no SUS em 2012, suscita a articulação interfederativa a fim de promover o acesso das PcD aos diferentes tipos de tratamentos especializados, mediante pactuações e governança regionais nas quais participam dirigentes de estados/municípios de uma determinada região de saúde. A garantia do direito ao tratamento odontológico e ao cuidado integral não é tarefa simples, e cobra uma articulação virtuosa entre os diferentes pontos de atenção da RCPD^9^, na qual deveria se destacar a APS como coordenadora dos cuidados à saúde^8^.

Estudo abrangendo 930 CEO mostrou que 85% estavam sob gestão municipal, e 10% não ofereciam atendimento à PcD, apesar de obrigatório. O acesso do usuário era referenciado pela APS em pouco mais da metade das unidades especializadas. Uma proporção expressiva (42,7%) possibilitava o agendamento por meio da demanda espontânea^10^, mas nenhum estudo investigou esta situação contrastante. Pode-se admitir que a implementação desta política pública depende de como organizações/profissionais da linha de frente atuam. Produzir informação científica sobre a interface entre os serviços de APS e especializados voltados à PcD pode ajudar na compreensão dos avanços e dos gargalos para a construção das redes integradas de saúde.

O objetivo deste trabalho é descrever a atuação dos profissionais e das organizações da linha de frente em relação à APS e às atividades de planejamento/avaliação, a fim de compreender o exercício da discricionariedade na implementação de diferentes formas de acesso à assistência odontológica especializada na RCPD em duas regiões de saúde brasileiras.

## MÉTODOS

Realizou-se um estudo de caso^11^ em duas regiões cujo acesso à assistência odontológica especializada era distinto. As regiões foram selecionadas com base em dados documentais e de entrevistas obtidos em um estudo mais amplo^a^, aprovado pelo Comitê de Ética em Pesquisa da Faculdade de Saúde Pública-USP, nº 3.441.243.

O acesso à assistência especializada era referenciado na região de saúde São José do Rio Preto, identificada como região A, enquanto na região Salvador (região B) era misto, ou seja, importante proporção dos usuários acessava livremente a unidade especializada sem necessidade de um encaminhamento/referência formal, sujeitando-se a regras próprias desta.

As duas regiões foram selecionadas intencionalmente, porque ambas tinham características socioeconômicas e de oferta de serviços semelhantes. Em tipologia realizada por Viana et al.^12^, foram classificadas como regiões com alta situação socioeconômica e alta oferta e complexidade dos serviços da saúde. Além do mais, elas contavam com a presença de centros especializados de reabilitação (CER), centros de especialidades odontológicas (CEO), serviços municipais e/ou filantrópicos de especialidades com ações de reabilitação ao usuário (Associação de Pais e Amigos dos Excepcionais, Associação de Assistência à Criança Deficiente, organizações não governamentais), e instituições formadoras (universidades, faculdades e cursos de residências médica e/ou multiprofissional).

A coleta de dados ocorreu entre julho e dezembro de 2019, feita por profissionais previamente treinados. Concordaram em participar da pesquisa cinco unidades que prestavam assistência odontológica especializada às PcD, e quatro unidades da APS sob gestão municipal das duas regiões. Das cinco unidades especializadas, quatro estavam sob gestão municipal (uma na região A e três na região B), e uma sob gestão estadual (região B). Foram entrevistados nove profissionais da linha de frente, representados neste estudo pelos gerentes, considerados atores-chaves dentro das organizações. Quando a unidade não possuía gerente, o informante-chave foi o cirurgião-dentista responsável pelo atendimento à PcD.

Instrumentos estruturados (questionários) e semiestruturados (roteiros) foram utilizados para a coleta dos dados. Neste estudo foram analisadas as respostas dos entrevistados relativas à frequência de acesso da PcD às unidades especializadas via APS, aos critérios de priorização que eram adotados, às dificuldades para que a APS fossem a principal porta de acesso aos serviços, às iniciativas da APS para exercer a coordenação do cuidado, e ao uso de instrumentos de monitoramento/avaliação, sua frequência e os atores participantes.

Relatórios técnicos e atas de reuniões colegiadas que continham conteúdo relativo à implementação da RCPD no nível loco-regional, abrangendo eventos de 1988 a 2019, foram examinados, buscando-se identificar dispositivos normativos e aspectos ligados à organização da APS e das atividades de planejamento/avaliação. Para isso, foram consultados decretos, leis e portarias relacionados ao tema nos sites institucionais das regiões (Secretarias Municipais e Estaduais, Secretaria de Estado de Planejamento e Gestão), relatórios técnicos produzidos no âmbito das regiões de saúde, e atas de reuniões de Comissão Intergestores Bipartite (CIB) e Comissão Intergestores Regional (CIR), disponibilizadas publicamente ou pelos técnicos dos serviços. A seleção dos trechos dos documentos se deu a partir de palavras-chaves que identificassem núcleos de sentido^13^ relacionados às seguintes categorias: tipo de acesso; atributo da APS relativo à coordenação do cuidado; planejamento, avaliação e monitoramento.

O acesso diz respeito às estratégias adotadas para que os usuários consigam utilizar os serviços de que necessitavam. A coordenação do cuidado refere-se à condução clínica dos casos por meio da articulação entre ações e serviços oferecidos por diferentes unidades da rede de atenção para responder às necessidades de saúde dos usuários, visando à reorientação do modelo assistencial. O planejamento, avaliação e monitoramento dizem respeito aos instrumentos e indicadores específicos que orientam o processo de trabalho dos profissionais da linha de frente, bem como a qualidade das ações ofertadas^8^.

## RESULTADOS

Os resultados são apresentados segundo as categorias temáticas e a região. Os Quadros 1, 2 e 3 apresentam trechos das entrevistas, agrupados nas categorias “acesso à assistência odontológica especializada para a PcD”, “APS como coordenadora dos cuidados”, “planejamento, avaliação e monitoramento”, respectivamente. O Quadro 4 apresenta a síntese dos resultados, por categoria temática, nas regiões A e B. Os entrevistados foram identificados como E_R1_ , E_R2_ e E_R3_ para a região A, e E_R4_ a E_R9_ para a região B.

**Quadro 4:** Síntese dos resultados segundo as categorias temáticas. RCPD no âmbito da saúde bucal (RCPD-SB), em duas regiões de saúde brasileiras, 2019.

Organização da RCPD-SB		
Categorias temáticas	Região A	Região B
Acesso	Apenas via referência da APS.	Referenciado pela APS ou outro serviço especializado ou livre demanda.
APS na coordenação dos cuidados	Sim. Realizavam matriciamento sobre o tema (RCPD), estabeleciam linhas de cuidado, ofertavam ações de reabilitação e conseguiam agendar, em tempo oportuno, consultas em reabilitação para usuários encaminhados.	Não coordenava, especialmente pela baixa cobertura populacional. A contrarreferência dos CEO para APS não acontecia.
Planejamento, monitoramento e avaliação	Avaliação e monitoramento mensais, em reuniões de equipe, com uso de indicadores e instrumento específico criado pela gerência CEO. Grupo condutor constituía dispositivo para compartilhamento de informações e deliberações.	Não fazia parte da rotina institucional dos serviços da RCPD-SB. Cada serviço definia seu processo de trabalho e nem sempre seguiam os padrões estabelecidos em portarias e políticas ministeriais. Ausência de espaço para compartilhamento das informações.

RCPD: Rede de Cuidados à Pessoa com Deficiência; APS: atenção primária à saúde; CEO: centros de especialidades odontológicas.

**Quadro 2:** Relatos dos entrevistados agrupados na categoria “APS como coordenadora dos cuidados” para as regiões A (acesso referenciado) e B (acesso misto).

Região A	Região B
“*A gente dá apoio matricial para a atenção básica, fazemos capacitações quatro vezes por ano. E isso é pactuado com a atenção básica,*” (E_R2_).	“*A atenção básica está bastante fragmentada. Tanto é que cada dia aumenta mais o meu número de diabéticos, acho que isso é um sinal grande da falha da atenção básica*” (E_R7_).
	“*Cobertura ela* [APS] *não tem no Salvador todo, em torno de 30%* […] *a gente queria muito que existisse a cobertura da atenção primária, que acho que resolveria muitos problemas nossos, se a gente conseguisse uma cobertura maior*” (E_R8_).
	“*Os especiais* [PcD]*, não. Eu resolvo tudo aqui. Eu nem devolvo, apenas os pacientes que não são especiais* [PcD], *que agora estão retornando para UBS*” (E_R8_).

APS: atenção primária à saúde; PcD: pessoa com deficiência; UBS: unidade básica de saúde.

**Quadro 3:** Relatos dos entrevistados agrupados na categoria “planejamento, avaliação e monitoramento” para as regiões A (acesso referenciado) e B (acesso misto).

Região A	Região B
“*Se você não conhece portaria, você não sabe como fazer o CEO funcionar. E tem portaria para tudo. É ridícula a produção que o Ministério exige, e tem município que não dá conta. Então é uma questão de conhecer portaria e falar: ‘agora vocês vão ter que fazer!’*” (E_R2_).	“*Não existe planejamento de ação*” (E_R9_).
“*Eu li a portaria, li com eles, discuti nas reuniões de equipe, levei as portarias, e eu acho que isso é importante. Passei as portarias para eles se apropriarem*” (E_R2_).	“*Pelo menos a cada 2 meses* [realizam reunião de equipe]*, por conta da carga horária dos profissionais (a equipe toda)*” (E_R8_).
	“*A gente atende paciente, mas não tem meta. Normalmente o CEO tipo III faz 190 procedimentos básicos que é do PNE, da qual pelo menos 50% têm que ser procedimentos restauradores*” (E_R8_).

CEO: centros de especialidades odontológicas; PNE: pessoas com necessidades especiais.

As discussões entre diferentes níveis de governo no espaço da CIB, sobre gestão e organização da RCPD tiveram início no final de 2011 na região A, enquanto na região B entraram em pauta somente a partir de 2013. Na região A, onde o acesso à assistência odontológica especializada ocorria apenas por meio de um pedido formal, expedido por uma unidade da rede de serviços, observou-se que esta referência acontecia quando o cirurgião-dentista da APS considerava não dispor das condições necessárias para prover o tratamento adequado. Assim ocorria também na região B, onde o acesso era por demanda espontânea e referenciada. Neste tipo misto, o usuário podia ingressar livremente, sem necessidade de um pedido formal, e de acordo com as regras próprias da unidade especializada.

Para acessar outros serviços especializados em reabilitação que compunham a RCPD (CER, AME), a UBS era a porta preferencial de acesso na região A, cabendo-lhe a referência de usuários aos serviços especializados. O acesso às unidades especializadas localizadas no município polo se dava via APS, obrigatoriamente. Casos que não obedecessem a este critério eram acolhidos para orientação e redirecionados para a APS.

Trechos de documentos mostraram que a necessidade de garantir a acessibilidade da RCPD era um tema recorrente nas reuniões da Comissão Intersetorial de Atenção à Pessoa com Deficiência, na região A. Este esforço significava maior disponibilidade de transporte sanitário e oferta de auxílio para uso de ônibus de linha comum.

Na região B, havia unidades especializadas de assistência odontológica administradas pelas esferas estadual e municipal. Observou-se que os profissionais vinculados à unidade estadual não se comunicavam nem interagiam com os dentistas da rede municipal que atuavam nas unidades de APS e nas especializadas. Na prática, aquele serviço possuía uma porta de entrada que seguia regras próprias pré-estabelecidas. Por outro lado, na região A foi reconhecida a importância do trabalho em rede para qualificar a assistência à PcD e, consequentemente, à RCPD (Quadro 1).

**Quadro 1:** Relatos dos entrevistados agrupados na categoria “acesso à assistência odontológica especializada para a PcD” para as regiões A (acesso referenciado) e B (acesso misto).

Região A	Região B
“*A* UBS *é a porta de entrada preferencial na região*” (E_R1_).	“*A porta de entrada é através de encaminhamento da* UBS. *Eles vão pra* UBS, *ou* PA*, ou* UPA*, ou* ESF *(quando coberto), e, caso o dentista não faça o atendimento, eles fazem um documento encaminhando o paciente para ser atendido no CEO*” (E_R4_).
“*Eu acho que, se a gente tem um CEO que funciona hoje, é porque a gente tem uma atenção básica que começou a trabalhar direito, está funcionando. Senão você não organiza CEO, se a atenção básica não encaminhar direito, protocolo.*” (E_R2_)	“*Então, os pacientes já sabem que, chegando aqui, com sua carteirinha do instituto onde ele é acompanhado, com seu relatório médico informando que é um portador de necessidades especiais, que ele vai ser atendido aqui, sem precisar de um encaminhamento*” (E_R5_).
“*Nós temos que trabalhar em rede, CEO, atenção básica, especializada. Só assim vai funcionar*” (E_R3_).	“*Para ele entrar na unidade, ele passa pelo* GO, *que vai dizer se é perfil ou não. Ele entra e aqui dentro ele passa por um grupo de profissionais que o avalia e vê a necessidade real do usuário, e pode colocá-lo como prioritário ou não*” (E_R6_).

O custo dos meios de transporte na região B, e a maneira como PcD auditiva eram recebidas nas unidades especializadas da região A foram identificados como possíveis barreiras de acesso aos serviços. O município polo da região A ofertava uma profissional intérprete da Língua Brasileira de Sinais (Libras), que atuava como tradutora durante a consulta odontológica especializada, mediante agendamento prévio.

Identificou-se que as UBS não eram a porta preferencial de acesso aos serviços de saúde para o usuário na região B. Seu papel como coordenadora dos cuidados mostrava-se fragilizado, em decorrência do baixo potencial de cobertura populacional e da fragmentação da atenção, apontando para o enorme desafio de integrar a APS com os serviços especializados. Em reuniões de CIB, foram propostas estratégias para elaboração do Plano Estadual, como estabelecimento de linhas de cuidado, ordenamento do fluxo, cadastramento de equipes de saúde da família com saúde bucal e expansão da APS. Entretanto, não foram observadas mudanças na linha de frente. A contrarreferência dos usuários à APS pelos profissionais das unidades especializadas da região B também não acontecia (Quadro 2).

Na região A, a APS era central no cuidado, sendo a principal responsável pela assistência às PcD. Os profissionais participavam de matriciamento sobre o tema, ofertavam ações de reabilitação e conseguiam agendar, em tempo hábil, consultas em reabilitação para os usuários encaminhados. A agenda de matriciamento do CEO com a APS era planejada anualmente em reunião de equipe. As UBS que possuíam equipes de saúde bucal, em ambas as regiões, realizavam atendimento às PcD. Aos CEO eram encaminhados os casos mais complexos, como os de usuários não colaborativos, com necessidade de contenção ou de uma intervenção mais individualizada.

Planejamento, avaliação e monitoramento das ações não faziam parte da rotina institucional dos serviços na região B. Como consequência, o processo de trabalho não era monitorado, nem orientado por critérios previamente estabelecidos. Somente alguns serviços realizavam reuniões periódicas de equipes e monitoramento de indicadores. Cabe destacar que o Plano Estadual da RCPD previa como diretriz para qualificação da gestão a produção, o acompanhamento e monitoramento das informações, além da qualificação profissional (Quadro 3).

Na região A, o planejamento, avaliação e monitoramento eram parte da rotina institucional dos serviços, e alguns gestores (como a gerente do CEO) desenvolveram instrumento próprio para avaliação e monitoramento de indicadores específicos relacionados, entre outros, à satisfação do usuário, ao número de procedimentos dentários restauradores, à proporção de absenteísmo, porcentagem de tratamento concluídos, número de exodontias, e quantidade de PcD atendidas em todas as especialidades. As reuniões do Grupo Condutor da RCPD também reconheciam a importância tanto da avaliação e monitoramento das ações para consolidação da RCPD, através do diagnóstico situacional (quantidade de PcD, principais demandas de saúde, elaboração do plano regional), quanto do papel da gerência no uso dos instrumentos de monitoramento das ações e compartilhamento das informações em reunião com toda a equipe.

## DISCUSSÃO

Neste estudo de caso, descreveu-se a atuação dos profissionais e das organizações da linha de frente, a fim de compreender o exercício da discricionariedade na implementação de diferentes formas de acesso à assistência odontológica especializada na RCPD em duas regiões de saúde brasileiras.

Os achados evidenciaram distintas características em relação à APS, às atividades de planejamento e avaliação e à estratégia de acesso adotada. Na região A, onde o acesso era referenciado, observou-se que a APS tinha papel de coordenação do cuidado, e o planejamento/avaliação faziam parte da rotina institucional dos serviços. Na região B, de acesso misto, notou-se que a troca de informação entre as unidades de APS e as unidades especializadas era esporádica, e que o papel de coordenação do cuidado não era um atributo das equipes de APS. Nesta região, as atividades de planejamento/avaliação não estavam incorporadas à rotina das organizações, conforme orienta a PNSB, pois cada serviço decidia, através dos seus implementadores, se as realizava ou não.

Com isso, o exercício da discricionariedade na forma pela qual o acesso do usuário era regulado em cada região estava estreitamente relacionado ao papel da APS na rede assistencial e às características das unidades especializadas quanto às atividades de planejamento/avaliação. Os distintos resultados de implementação encontrados neste estudo de caso confirmaram a ideia de que a implementação efetiva é sempre realizada com base nas referências que os implementadores de fato adotam para desempenhar suas funções, e corroboram a noção de que o exercício da discricionariedade encerra diferentes dimensões voltadas à obtenção de resultados política e socialmente desejados, em uma direção legitimamente definida conforme o ambiente relacional e institucional presente no contexto loco-regional, sobressaindo-se tanto a dimensão administrativa quanto a dimensão política^3^.

A discricionariedade administrativa é entendida como o uso de estratégias para introduzir mudanças procedimentais^3^. Neste estudo de caso, diz respeito ao uso de canais de comunicação e fluxos compartilhados com auxílio de protocolos e outros instrumentos comuns, a fim de promover a articulação entre as unidades de APS e os serviços odontológicos especializados, com vistas a obter níveis crescentes de integralidade do cuidado. Inclui também a decisão de criar espaços de planejamento e avaliação compartilhados entre gerentes de diferentes programas e serviços mantidos dentro de uma mesma organização, ou envolvendo diferentes unidades de igual ou de distinta densidade tecnológica, uma vez que o uso da ação discricionária dos atores públicos requer o desenvolvimento de habilidades de governança^3^. Na região A, a implementação de protocolos e fluxos comuns a toda a rede foi uma característica que auxiliou a articulação das organizações de nível primário e especializado, permitindo a adoção do acesso referenciado pelos serviços odontológicos especializados.

A dimensão política diz respeito aos valores e aos referenciais em jogo na interação dos atores e à competência de combiná-los durante o exercício de poder para torná-los funcionais à obtenção dos fins desejados^3^. Embora seja possível identificar um componente relacionado à trajetória individual dos atores, estes referenciais não são apenas produzidos por escolhas individuais, mas sim engendrados pelas influências derivadas das relações estabelecidas no curso da implementação em contextos institucionais e relacionais específicos^1^, nos quais a colaboração entre as partes interessadas pode encurtar o caminho para o alcance dos desfechos pretendidos^14^.

Na região A, uma política de interesse geral relacionada à construção de uma rede de cuidados integrados de saúde estava em curso, sendo mencionada tanto pelos entrevistados das unidades de APS quanto das unidades especializadas. Por outro lado, na região B essa construção não pertencia à perspectiva dos atores. O material obtido mostrou que a comunicação e a interação entre os profissionais destas unidades não eram recursos estruturantes das ações de cuidado. Neste ambiente relacional, as unidades operavam em uma lógica de prestação de serviços básicos e especializados isolada e fragmentada.

Após a aprovação das diretrizes da PNSB, em 2004, mais de mil CEO foram criados no Brasil para possibilitar o acesso referenciado das PcD e da população, em geral, a cuidados complexos, atendendo ao princípio da integralidade^15^. No que pese a estratégia por livre demanda ser considerada negativa como forma de acesso ao serviço especializado dentro de uma rede de atenção voltada à produção de cuidados integrados, ela pode ser interpretada positivamente pelo usuário que não precisa recorrer à unidade de APS nas consultas subsequentes. Em um contexto relacional e institucional no qual as unidades não operam de forma colaborativa, nem estabelecem compromissos recíprocos, e em que práticas fragmentadas predominam, recorrer a outra unidade pode gerar custos, incerteza e estresse para o usuário. Do ponto de vista da política pública sob análise, gera distorções na implementação dos CEO, criando condições para que sua operação passe a responder a lógicas de “guichê” voltadas à obtenção de resultados política e socialmente desejados, relacionados a interesses específicos de curto prazo, que podem ensejar uma perspectiva clientelística^16^.

Cabe destacar que, no Brasil, desde o final dos anos 1990, o nível local é responsável pela provisão dos serviços de APS, sendo a provisão de serviços especializados dependente de arranjos regionais entre os níveis municipal e estadual. Sistemas descentralizados em repúblicas federativas, à semelhança de países como Canadá e Austrália, podem representar uma vantagem importante, ao possibilitarem a contenção de custos para o nível central e darem maior autonomia e responsabilidade aos governos locais perante as necessidades de saúde da população. Mas é também um desafio porque, os municípios e as regiões ficam sujeitos a variações jurisdicionais, fragmentações na coordenação, cooperação e compartilhamento das informações^17^.

Examinando a atuação de agentes comunitários de saúde^6^, verificou-se que as atividades diferiam bastante, embora exercessem a mesma função e fossem regidas por uma mesma política. Além dos fatores individuais, aspectos organizacionais e de contexto exerceriam influência sobre o tipo de atividade desenvolvida pelos agentes em sua rotina. No presente estudo, a despeito dos serviços serem providos por profissionais da linha de frente, com formações ligadas à área da saúde e regidos por interesses gerais definidos pelas diretrizes de uma política pública voltada à implementação da RCPD, as estratégias de acesso à assistência odontológica especializada eram distintas. Apesar de semelhanças socioeconômicas e na oferta de serviços, a resposta da implementação não foi a mesma, sugerindo que o ambiente relacional e institucional exerce um papel importante.

Em relação às limitações deste estudo, é preciso destacar que a implementação na linha de frente é influenciada por múltiplas forças, muitas vezes concorrentes no sistema de implementação^14^. A opinião dos usuários dos serviços não foi incluída na análise deste trabalho, e investigações que incorporem tanto a visão dos profissionais quanto dos usuários poderiam complementar e aprofundar as considerações aqui levantadas, a fim de explorar efeitos da discricionariedade que poderiam gerar inclusão, equidade, exclusão e desigualdade. Diferenças socioculturais, da prevalência de PcD e de número de usuários em cada região também não foram consideradas. Apesar destes pontos e dos resultados serem provenientes de um estudo de caso, a investigação inova ao fazer uso da teoria de implementação de política pública numa perspectiva *bottom-up*, e explora domínios analíticos pouco investigados concernentes aos aspectos relacionais e institucionais que exerceriam influência na linha de frente da política pública aqui investigada.

Conclui-se que a implementação da política de assistência odontológica especializada à pessoa com deficiência está sujeita ao poder discricionário dos profissionais e das organizações da linha de frente, sugerindo que o ambiente relacional e institucional joga um papel importante no processo de implementação de políticas públicas em um sistema descentralizado e regionalizado de saúde, no qual diferentes estratégias de acesso aos serviços especializados estão associadas ao papel coordenador da APS e à condução de atividades de planejamento/avaliação voltadas à construção de uma rede de cuidados integrados de saúde.

## Footnotes

a “Desafios da implementação da Rede de Cuidado à Pessoa com Deficiência em diferentes contextos regionais: abordagem multidimensional e multiescalar” (MS-SCTIE-DECIT/CNPQ nº 442801/2018-1).
